# DFT Study of MAX Phase Surfaces for Electrocatalyst Support Materials in Hydrogen Fuel Cells

**DOI:** 10.3390/ma14010077

**Published:** 2020-12-25

**Authors:** Jonathan Gertzen, Pieter Levecque, Tokoloho Rampai, Tracey van Heerden

**Affiliations:** 1 HySA/Catalysis Centre of Competence, Catalysis Institute, Department of Chemical Engineering, University of Cape Town, Cape Town 7700, South Africa; grtjon001@myuct.ac.za (J.G.); pieter.levecque@uct.ac.za (P.L.); 2 Department of Chemical Engineering, University of Cape Town, Cape Town 7700, South Africa; tokoloho.rampai@uct.ac.za; 3 Catalysis Institute, Department of Chemical Engineering, University of Cape Town, Cape Town 7700, South Africa

**Keywords:** MAX phases, surfaces, density functional theory, electrical conductivity, hydrogen fuel cells, Ti_3_SiC_2_, Ti_3_AlC_2_, Ti_2_AlC, BoltzTraP2

## Abstract

In moving towards a greener global energy supply, hydrogen fuel cells are expected to play an increasingly significant role. New catalyst support materials are being sought with increased durability. MAX phases show promise as support materials due to their unique properties. The layered structure gives rise to various potential (001) surfaces. DFT is used to determine the most stable (001) surface terminations of Ti_2_AlC, Ti_3_AlC_2_ and Ti_3_SiC_2_. The electrical resistivities calculated using BoltzTraP2 show good agreement with the experimental values, with resistivities of 0.460 µΩ m for Ti_2_AlC, 0.370 µΩ m for Ti_3_AlC_2_ and 0.268 µΩ m for Ti_3_SiC_2_. Surfaces with Al or Si at the surface and the corresponding Ti surface show the lowest cleavage energy of the different (001) surfaces. MAX phases could therefore be used as electrocatalyst support materials, with Ti_3_SiC_2_ showing the greatest potential.

## 1. Introduction

MAX phases are a relatively new class of materials of the form M_*n*+1_AX_*n*_, where M is an early transition metal, A is a group III or IV A-group element and X is either C or N. They exist in different ratios, with n from one to three [1]. MAX phases possess a unique layered structure, which gives them the properties of both metals and ceramics [2,3]. Initially discovered in the 1960s [4,5,6,7,8], they only gained significant interest in 1996 with the synthesis of single-phase Ti_3_SiC_2_ [9]. Certain MAX phases show properties from metals, such as being fracture tough, electrically and thermally conductive and being relatively soft and machinable, as well as from ceramics, such as showing oxidation resistance, thermal shock resistant and elastically stiff, among other properties [2,10].

Hydrogen fuel cells, predominantly using a proton exchange membrane (PEM), require catalyst support materials with good electrical conductivity and good oxidation resistance. For commercialisation, a replacement of the carbon black support material, which experiences corrosion under high operating potentials, needs to be found to to increase the proton exchange membrane fuel cell’s (PEMFC) durability [11]. MAX phases show good oxidation resistance along with good electrical conductivity; therefore, they could be an option as the catalyst support material.

However, due to the layered structure of the MAX phases, different (001) surface terminations are possible. Since a catalyst support material would have surfaces present on a particle, the most stable surfaces were investigated. Other studies have investigated the (001) surfaces of some MAX phases [12,13,14,15,16,17]; however, there is little consistency in the literature.

The earliest study of MAX phase (001) surfaces was by Sun and Ahuja [12], who studied Cr_2_AlC. They found that the Al termination was most stable. Music et al. [13,14] then studied other 211 MAX phases including Ti_2_AlC, confirming that the A-group termination was the most stable (001) surface. However, Music et al. [13,14] did not distinguish between surfaces for which it is possible to have different elements in the subsurface layer. For Ti_2_AlC, they only reported the surface energy with Ti on the surface, but did not mention whether Al or C was in the subsurface layer. The same authors also did not report what they used as their reference energies, which makes verification of the accuracy of their results quite challenging.

Wang et al. [15] included the different subsurface atoms in their naming of surface terminations, along with noting the pitfalls of using symmetric slabs without stoichiometric reference systems. Their conclusion of the Al(Ti) and Ti(C) surfaces being most stable agrees with earlier studies though.

Zhang and Wang [16] studied the (001) surfaces of Ti_3_AlC_2_ and Ti_3_SiC_2_ using 1 × 1 unit cells. They correctly differentiate between the six different surfaces possible and calculated the surface energies of each surface. They used the symmetric slabs to calculate the surface energy, with changes in elemental chemical potential as reference energies. The most stable surface depended on the change in chemical potential, with Ti2(C) and Si(Ti2) for Ti_3_SiC_2_ and Al(Ti2) and Ti2(C) for Ti_3_AlC_2_ being the most stable surfaces, in that order. The challenge with the approach of Zhang and Wang [16] is that creating a symmetrical slab for a MAX phase (001) surface requires the system to be non-stoichiometric. Therefore, the bulk energy cannot be used in the calculation of the surface energy, and the chemical potential of the pure element is used instead. This is quite a significant assumption to make since it implies that the chemical potential of the elemental system is the same as in the surface system; especially in the case of MAX phases. where the layered structure changes the surrounding environment of each element completely.

Orellana and Gutiérrez [17] studied the (001) surfaces of Ti_3_SiC_2_ using DFT and molecular dynamics, concluding that at low temperatures, Si(Ti2) and Ti2(Si) were most stable, while at high temperatures, Ti1(C) and Ti2(C) were most stable. They commented that surfaces with C terminations were least stable.

Point defect vacancies have been more extensively studied in the literature for Ti_2_AlC [18,19,20,21] and for Ti_3_AlC_2_ and Ti_3_SiC_2_ [22,23,24,25,26]. There is consensus that the A-group atom in MAX phases has the lowest vacancy formation energy, which agrees with the experimental results [27,28,29,30] showing that oxides of the A-element form on the surface during oxidation, e.g., Al_2_O_3_ and SiO_2_.

Through the design of this study, it was ensured that some of the inconsistencies described were addressed and/or improved. A differentiation was made between surfaces with different atoms in the sub-surface layer, and the reference energy was calculated from the bulk systems to then use in calculating the cleavage energy.

Furthermore, in reporting these results, cleavage energy as opposed to surface energy is shown. The authors have the view that the following assumption of Wang et al. [15] is inaccurate: the difference between the unrelaxed cleavage energy and the relaxation energy equates to the surface energy. This is because the chemical potential of an element is not representative of the chemical potential of the corresponding element in the MAX phase structure.

Taking the above considerations into account, in this study, we evaluated three MAX phases, Ti_2_AlC, Ti_3_AlC_2_ and Ti_3_SiC_2_ (See Figure S1 and Table S1 for the unit cells and lattice parameters), for their suitability as electrocatalyst support materials. The key properties investigated were electrical conductivity, determined through Boltzmann transport equations, and the determination of the most stable (001) surface terminations.

## 2. Theoretical Method

Density functional theory (DFT) [31,32] calculations were performed using the Vienna Ab-initio Simulation Package (VASP) [33]. Projector augmented-wave (PAW) [34] pseudopotentials were used with the generalised gradient approximation (GGA) [35] Perdew–Burke–Ernzerhof (PBE) [36] functional. Four different functionals were investigated, namely the local density approximation (LDA) [37], GGA with Perdew–Wang 1991 (PW91) [38], PBE and Revised Perdew–Burke–Ernzerhof (RPBE) [39]. Of these four, the PBE functional was determined to be most accurate for these systems (see the Supplementary Information). The PBE functional is also consistent with recent literature on MAX phases. k-point sampling according to the Monkhorst–Pack scheme [40,41] was used to optimise the k-point grid for each MAX phase (Table S2). The electronic convergence criteria were set at $1\times 10^{-4}$ eV and the force convergence criteria at 0.03 eV/Å. The cut-off energy was optimised for each MAX phase (Table S3). Both the *a* and *c* lattice parameters of each bulk unit cell were optimised (Figure S2, Table S4) and the Birch–Murnaghan [42] equation-of-state fitted to calculate the bulk modulus (Figure S3). The open source software pymatgen [43] was used to fit the Birch–Murnaghan EOS and calculate the bulk modulus, which showed good agreement with the experimental values from Barsoum [44] and Hettinger et al. [45]. Bader charges [46,47,48,49] were calculated for each atom and the charge density visualised using VESTA [50,51].

A (2 × 2) supercell was used for all surface calculation. The k-point grid, cut-off energy, vacuum gap and number of atomic layers required for energy convergence were optimised for each surface system, with the smallest common parameters utilised for each MAX phase. A 2nd order Methfessel–Paxton smearing scheme [52] with a width of 0.1 eV was used. Surface slabs were optimised under total atomic relaxation until the centre layers resembled bulk characteristics, from which the slabs were halved and the bottom layers fixed in position. The cleavage energy was calculated based on the method by Lu et al. [53], fitting the slab number and the number of surface atoms with total system energy to calculate the cleavage energy and fitted bulk reference energy.

To calculate the electrical conductivity of the MAX phases and the surfaces, the program BoltzTraP2 [54,55] was used. Using a constant relaxation time approximation of $3\times 10^{-14}$ s ([1], p. 179), the electrical conductivity was calculated.

## 3. Results and Discussion

### 3.1. Bulk Properties

Before surface properties were determined, the bulk systems were calculated and the lattice parameters and bulk moduli compared against experimental and DFT results from other authors. The results are shown in Table 1. The results are in excellent agreement with other DFT results and in good agreement with the experimental measurements.

It is well documented in the literature that the GGA-PBE functional tends to overestimate lattice parameters whilst underestimating the bulk modulus [60]; therefore, since the calculated lattice parameters are larger than experimental values whilst the bulk moduli are mostly smaller, the results are within the expected range for calculations from the PBE functional. Whilst more accurate meta-GGA functionals are available, utilising the PBE functional was deemed sufficient in order to report on the novel vacancy defect formation energies, the cleavage energies of different (001) surfaces and the calculated electrical resistivities of the MAX phases.

### 3.2. Electronic Properties

Different electronic properties were calculated for the bulk unit cells, including the density of states, charge density, Bader charge analysis and the electrical conductivity from Boltzmann transport coefficients. These are presented below.

#### 3.2.1. Density of States

**Figure 1:**
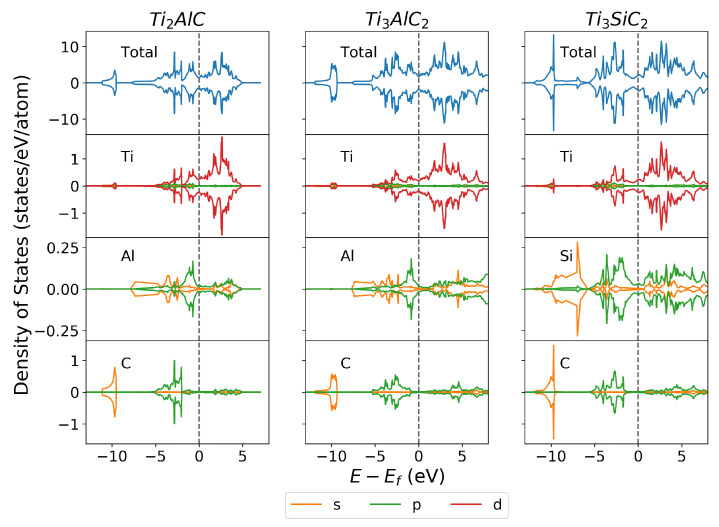
Element and orbital resolved density of states for Ti_2_AlC, Ti_3_AlC_2_ and Ti_3_SiC_2_. The Fermi level is shown by the black dashed line.

The local, elemental-resolved density of states (DOS) of the bulk unit cells was calculated to investigate the bonding between atoms. Additionally, a continuous DOS across the Fermi level gave an indication that the material is electrically conductive. Figure 1 shows the total DOS and the elemental and orbital resolved DOS of the bulk unit cell. The total DOS has units of states/unit cell, while the orbital resolved elemental DOS has units of states/(eV.atom). These DOS results show the agreement of these calculations with the experimental and computational literature [44,61,62,63].

The continuous total DOS across the Fermi level agrees with experimental literature [44,61,62,63] showing good electrical conductivity. It can be seen that the biggest contributor to the continuous DOS at the Fermi level is the Ti 3d orbital. Since TiC is not conductive due to the covalent bonds between Ti and C, it could be expected that the Al or Si would primarily contribute to the continuous DOS at the Fermi level. However, Figure 1 shows that the orbitals of Al and Si are not the primary contributors to the DOS above the Fermi level. The total DOS more closely follows the pattern of the Ti 3d orbital, showing the the Ti 3d orbital contributes primarily towards the DOS above the Fermi level. This shows that the presence of Al and Si modifies the electronic structure of Ti such that the structure becomes electrically conductive.

While Al and Si modify the electronic structure of Ti, the bond between Ti and C remains strong. There is hybridisation between the Ti 3d and the C 2s orbital around −10 eV indicating a strong bond, in addition to hybridisation between the Ti 3d orbital and the C 2p orbital closer to the Fermi level. These show the strong covalent bonds between Ti and C atoms within the MAX phase structure. Additionally, the Ti 3d to Ti 3d bond occurs around the Fermi level, showing the general trend of metallic M—M bonds in MAX phases.

The Ti 3d orbital also shows hybridisation with the Al 3p and Si 3p orbitals. However, this hybridisation is closer to the Fermi level, indicating a weaker bond compared with the Ti-C bond. The Ti-Si bond in Ti_3_SiC_2_ is at a slightly lower energy than the Ti-Al bond in Ti_3_AlC_2_, suggesting that the Ti-Si bond is stronger than the Ti-Al bond. This stronger Ti-Si bond explains why the *c* lattice parameter of Ti_3_SiC_2_ is shorter than Ti_3_AlC_2_, having the same atomic structure. As will be shown later, this also has an effect on the cleavage energy of surfaces cleaved at the Ti-Si and Ti-Al bond.

#### 3.2.2. Charge Density and Bader Charge Analysis

**Figure 2:**
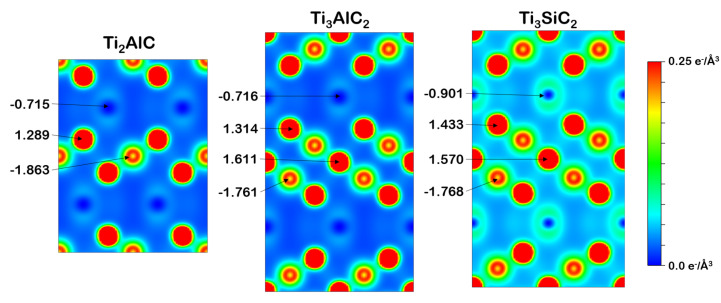
Charge density colour map through a (110) slice of the bulk unit cell with the Bader charge of each element.

The charge density of the bulk unit cells is useful for determining areas of high and low electron density. Shown in Figure 2 is a slice through the (110) plane of the bulk unit cell. The colour map goes from blue (0 e^−^/${Å}^{3}$) to red (0.25 e^−^/${Å}^{3}$) (an additional greyscale version can be found Figure S4). Bader charge analysis was performed on the bulk unit cell to empirically determine the charge distribution between elements. The Bader charge was calculated using the number of valence electrons used in the DFT simulation, as well as the number of electrons assigned to each atom through Bader charge analysis, shown in Equation (1).

(1) Badercharge = Atomicvalenceelectrons − Baderelectrons

The highest charge density is around the Ti atoms due to the high number of valence electrons of each atom, while there is a lower density around the Al and Si atoms. The number of Ti and C layers has little effect on the Bader charge of the Al atoms in Ti_2_AlC and Ti_3_AlC_2_, shown by almost identical Bader charges of −0.715 and −0.716, respectively. However, the Bader charge of Si in Ti_3_SiC_2_ is more negative than that of Al in Ti_3_AlC_2_. The Si therefore draws more electrons from the surrounding Ti atoms than Al, which agrees with the stronger Ti-Si bond observed in the DOS, specifically the hybridisation between the Si 2p and Ti 3d at lower energy than the Al 2p and Ti 3d hybridisation.

Looking at how the Bader charge on the C atom varies between MAX phases, the Bader charge in Ti_2_AlC is more negative than in Ti_3_AlC_2_ or Ti_3_SiC_2_. However, the Bader charge on C does not change significantly from Ti_3_AlC_2_ to Ti_3_SiC_2_. Therefore, it is primarily the stoichiometry of the MAX phase that affects the X atom Bader charge, not the identity of the A atom.

#### 3.2.3. Vacancy Formation Energy

The vacancy formation energy for the bulk MAX phases was investigated. A 2 × 2 × 1 supercell was used, with a single Al/Si atom removed. The reference energy for elemental Al and Si was calculated using bulk aluminium and bulk silicon, with the k-point grid, cut-off energy, and *a* lattice parameter optimised individually. The calculated vacancy formation energy is shown in Table 2. The energies are in good agreement with the calculations in the literature [18,22,25]. The results suggest that removing a Si atom from Ti_3_SiC_2_ requires less energy than removing an Al from Ti_3_AlC_2_, while removing an Al atom from Ti_2_AlC and Ti_3_AlC_2_ requires similar energy.

Wang et al. [18], using the Cambridge Serial Total Energy Package (CASTEP) code, the GGA-PW91 functional and a smaller 2 × 2 × 1 supercell, reported a mono-vacancy formation energy of 2.73 eV for Al in Ti_2_AlC. A mono-vacancy in a smaller supercell results in a stoichiometry of Ti_2_Al_0.875_C, while this study, using a 2 × 2 × 2 supercell, results in a stoichiometry of Ti_2_Al_0.9375_C. Additionally, the PW91 functional generally results in larger bond lengths, and therefore less strong bonds. Given these calculation parameter differences, an increase of only 5.57% in the vacancy formation energy shows good agreement.

Calculations from Wang et al. [22] and Zhang et al. [25] for Ti_3_AlC_2_ and Ti_3_SiC_2_ show good agreement for Ti_3_SiC_2_, with both reporting mono-vacancy formation energies of 2.1 eV for a Si defect. Wang et al. [22] reported a value of 2.2 eV for an Al mono-vacancy in Ti_3_AlC_2_, which is less of an agreement. This may be due to the use of a 2 × 2 × 1 supercell by both authors. However, the trend of the energy required to remove an Al atom from Ti_3_AlC_2_ being larger than for removing a Si from Ti_3_SiC_2_ still holds.

#### 3.2.4. Electrical Conductivity

**Figure 3:**
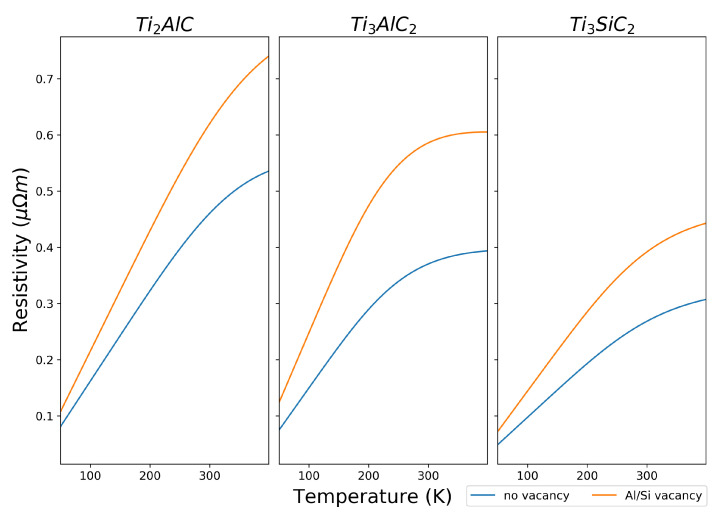
Resistivity of bulk MAX phases against temperature with and without an Al/Si vacancy from BoltzTraP2. Resistivities are taken at the chemical potential closest to zero, specifically at a values of $\mu -E_{f}$ of 0.054 eV and 0.058 eV for Ti_2_AlC, −0.024 eV and −0.036 eV for Ti_3_AlC_2_ without and with an Al vacancy, respectively, and 0.036 eV and 0.022 eV for Ti_3_SiC_2_, without and with a Si vacancy, respectively.

DFT calculations in and of themselves cannot provide a value for electrical conductivity, and comparisons can only be made by looking at the difference between the number of states at the Fermi level. The Boltzmann transport coefficients provide a route to calculate the electrical resistivity by interpolating the band structure of the material. The electrical resistivity at the chemical potential closest to zero relative to the Fermi level over a range of temperature was calculated using the constant relaxation time approximation using the BoltzTraP2 program [54,55] and is shown in Figure 3. Since BoltzTraP2 calculates the resistivity at discrete chemical potentials, for each MAX phase, the chemical potential closest to the Fermi level was determined from the data, of which the values are included in the figure caption.

At 300 K, the calculated electrical resistivities for Ti_2_AlC, Ti_3_AlC_2_ and Ti_3_SiC_2_, respectively, were 0.460 μΩ m, 0.370 μΩ m and 0.268 μΩ m. These are in good agreement with experimental electrical resistivities measured by Magnuson and Mattesini [61] (0.44 μΩ m for Ti_2_AlC, 0.5 μΩ m for Ti_3_AlC_2_ and 0.25 μΩ m for Ti_3_SiC_2_), Scabarozi et al. [62] (0.36 μΩ m for Ti_2_AlC and 0.353 μΩ m for Ti_3_AlC_2_), Barsoum [44] (0.23 μΩ m for Ti_3_SiC_2_), and Wang and Zhou [63] (0.287 μΩ m for Ti_3_AlC_2_). The resistivity for all three MAX phases decreases as temperature increases; however, the resistivity for Ti_3_AlC_2_ seems to plateau beyond 300 K, indicating that the electrical conductivity should remain relatively consistent in higher temperature ranges.

These resistivities correlate to electrical conductivity values of $2.17\times 10^{4}$ S cm^−1^ for Ti_2_AlC, $2.70\times 10^{-4}$ S cm^−1^ for Ti_3_AlC_2_ and $3.73\times 10^{-4}$ S cm^−1^ for Ti_3_SiC_2_. Traditionally, carbon is used as the support material for PEMFCs, which has a reported conductivity of 4.0 S cm^−1^ [64]. However, carbon has low oxidation resistance [11]. On the other hand, tungsten carbide (WC) has high oxidation resistance and high electrical conductivity of $1.26\times 10^{-3}$ S cm^−1^ [65]; however, it is unstable above potentials of 0.8 V [11].

When an A-group atom mono0vacancy is created in a 2 × 2 × 2 supercell, the electrical resistivity increases for all three MAX phases. At 300 K, the calculated electrical resistivities for Ti_2_AlC, Ti_3_AlC_2_ and Ti_3_SiC_2_ with an A-group atom mono-vacancy, respectively, were 0.620 μΩ m, 0.586 μΩ m, and 0.392 μΩ m. While the electrical conductivity decreases with the removal of an A-group atom from the unit cell, the conductivity is within the same order of magnitude; therefore, this suggests that these MAX phases could be used in applications where electrical conductivity is required. These MAX phases could therefore be promising electrocatalyst support materials for hydrogen fuel cells, provided that other parameters such as surface area are met.

### 3.3. Cleavage Energies

Different (001) surfaces were cleaved from a 2 × 2 × S supercell of each MAX phase, where S depends on the number of atomic layers required to maintain stoichiometry, denoted as the number of slabs required. Depending on the offset from the origin of the unit cell, different surface terminations are possible. These were named according to both the surface and subsurface atomic layer, with the subsurface layer in brackets. The layered structure prevents symmetrical surfaces from being cleaved, since off-stoichiometric bulk reference unit cells are not possible. Additionally, it is not accurate to use the chemical potentials of pure elements since the electronic structure of each element is altered to such an extent that it does not share enough characteristics with the pure element to provide accurate reference energies. Stoichiometric unit cells were therefore used, which creates different surface terminations on the top and bottom surfaces. Due to the symmetry of the atomic layers, each surface is duplicated at the bottom of another surface unit cell. This requires half of the surfaces to be calculated, with the mirror surface duplicated on the underside of the slab. The cleavage energy of each surface was calculated and is shown in Table 3. For Ti_3_AlC_2_ and Ti_3_SiC_2_, Ti1 is between two C layers, while Ti2 sees a C layer on one side and an Al/Si layer on the other side.

The cleavage energies of Al(Ti) in Ti_2_AlC and Al(Ti2) in Ti_3_AlC_2_ are close, suggesting that the inclusion of additional Ti and C layers does not influence the Ti-Al bond strength, which agrees with the similar Bader charges seen in Figure 2. Similarly, the cleavage energy of Si(Ti2) in Ti_3_SiC_2_ is larger than Al(Ti2) in Ti_3_AlC_2_, agreeing with the stronger Si-Ti2 bond compared to the Al-Ti2 bond. For all three MAX phases, the surface with the A-group atom at the surface and the corresponding mirror surface show the lowest cleavage energy of the different surfaces, suggesting that the surfaces with the A-group atom at the surface are the most stable surfaces. The mirror surface has Ti or Ti2 present at the surface, which presents a complete Ti-C layered structure at the surface of the slab, while in other surfaces, this Ti-C structure is fully or partially broken. This suggests that the stability of a surface depends on whether the Ti-C structure remains intact. Surfaces where this is not the case have higher cleavage energies than surfaces where this is the case.

#### Surface Bader Charge Difference

**Figure 4:**
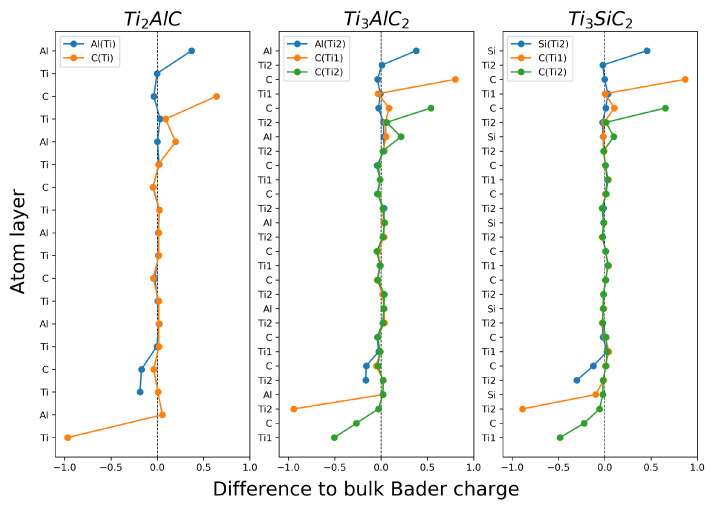
Bader charge difference for each surface of Ti_2_AlC, Ti_3_AlC_2_ and Ti_3_SiC_2_ compared to the corresponding bulk Bader charge. The Bader charge differences are aligned to their atomic layer.

When surfaces are cleaved, the electronic structure of the surface atoms changes due to bond breaking. This in turn affects the atomic layers beneath the surface. The difference between the Bader charge of the surface systems and of the bulk unit cells was calculated and is shown in Figure 4. Surface systems consisting of two full unit cells, or four stoichiometric slabs, in the direction perpendicular to the surface are shown in Figure 4 to show the convergence to bulk-like characteristics in the middle of the surface slab.

It can be seen that the Bader charge has the greatest magnitude of change near the surface, predominantly in the outer three layers. Surfaces with C, Al or Si at the surface show a positive change in Bader charge, indicating that they become more positive, while Ti surface atoms show a decrease, becoming more negative. The positive change in the Bader charge of C, Al and Si shows that they are unable to draw as many electrons towards themselves as they do in the bulk, which is understandable given the broken surface bonds. However, the Bader charge of C at the surface is larger than of Al or Si at the surface, indicating that C surface atoms experience a much greater change in environment when at the surface.

Surfaces with lower cleavage energies (Al(Ti) for Ti_2_AlC, Al(Ti2) for Ti_3_AlC_2_ and Si(Ti2) for Ti_3_SiC_2_) show a smaller change in Bader charge, both on the top and bottom surface. Additionally, for lower cleavage energy surfaces, the change in Bader charge resembles the bulk-like closer to the surface, affecting fewer atomic layers beneath the surface. This agrees well with the idea that surfaces with the Ti-C structure at or near the surface remaining intact yield more stable surfaces. Conversely, surfaces with a fully or partially broken Ti-C structure at the surface show a larger change in the difference to atomic bulk Bader charge, agreeing with the larger cleavage energy for those surfaces. Atomic layers at the surface that have a negative Bader charge in the bulk system show an increase in Bader charge, while those with a positive Bader charge (only Ti) show a decrease in Bader charge at the surface.

This suggests that the most stable (001) surfaces of MAX phases are those where the Ti-C structure is kept intact at or near the surface. This occurs for Al(Ti) and Ti(C) in Ti_2_AlC, Al(Ti2) and Ti2(C) for Ti_3_AlC_2_ and Si(Ti2) and Ti2(C) for Ti_3_SiC_2_.

## 4. Conclusions

In this study, two aspects of the properties of three MAX phases are studied, namely the electrical conductivity determined through the Boltzmann transport equations and the determination of the most stable (001) surface terminations. The three MAX phases investigated are Ti_2_AlC, Ti_3_AlC_2_ and Ti_3_SiC_2_. The electrical resistivities are calculated to be 0.460 μΩ m for Ti_2_AlC, 0.370 μΩ m for Ti_3_AlC_2_ and 0.268 μΩ m for Ti_3_SiC_2_ at 300 K, which show good agreement with the experimental resistivities. It is also seen that the electrical resistivity increases when an A-group mono-vacancy is formed, indicating that during the oxidation of the MAX phases, the electrical conductivity will decrease. However, since the electrical resistivities with an A-group mono-vacancy are of the same order of magnitude, the electrical resistivity will not decrease catastrophically during oxidation. In conducting surface stability calculations, the cleavage energy and change in Bader charge through the surface slab compared to the bulk system are determined. It is seen that surfaces with A-group terminations, i.e., Al(Ti), Al(Ti2) and Si(Ti2) for Ti_2_AlC, Ti_3_AlC_2_ and Ti_3_SiC_2_, respectively, along with the cleaved surface pair with a Ti termination, i.e., Ti(C), Ti2(C) and Ti2(C), respectively, are determined to be most stable. Due to these properties, MAX phases could be considered for electrocatalyst support materials in hydrogen fuel cells, with Ti_3_SiC_2_ showing the greatest promise.

## Figures and Tables

**Table 1:** Lattice parameters in Å and bulk modulus in GPa compared to theoretical and experimental values from the literature.

MAX Phase	*a*	*c*	Bulk Modulus	Type	Source
Ti_2_AlC	3.070	13.762	138.4	DFT	this work
	3.067	13.75	136	DFT	[56]
	3.052	13.64	-	Experimental	[57]
	-	-	144	Experimental	[45]
	3.04	13.6	-	Experimental	[3]
Ti_3_AlC_2_	3.075	18.678	158.3	DFT	this work
	3.083	18.66	156	DFT	[56]
	3.075	18.58	-	Experimental	[7]
	3.075	18.58	165	Experimental	[3]
	3.075	18.578	165	Experimental	[44]
Ti_3_SiC_2_	3.0665	17.865	193.8	DFT	this work
	3.077	17.715	192.61	DFT	[58]
	3.068	17.67	-	Experimental	[59]
	3.0665	17.671	185	Experimental	[44]
	3.066	17.671	-	Experimental	[4]

**Table 2:** Vacancy formation energy in eV of an A-group atom in bulk MAX phases.

MAX Phase	Vacancy Formation Energy
Ti_2_AlC	2.882
Ti_3_AlC_2_	2.812
Ti_3_SiC_2_	2.167

**Table 3:** Cleavage energies in eV/unit cell of each surface for each MAX phase.

MAX Phase	Surface	Mirror Surface	Cleavage Energy
Ti_2_AlC	Al(Ti)	Ti(C)	1.924
	C(Ti)	Ti(Al)	5.254
Ti_3_AlC_2_	Al(Ti2)	Ti2(C)	1.909
	C(Ti1)	Ti2(Al)	6.480
	C(Ti2)	Ti1(C)	4.639
Ti_3_SiC_2_	Si(Ti2)	Ti2(C)	2.802
	C(Ti1)	Ti2(Si)	6.479
	C(Ti2)	Ti1(C)	5.143

## Data Availability

The data presented in this study are available on request from the corresponding author.

## Supplementary Materials

The following are available online at https://www.mdpi.com/1996-1944/14/1/77/s1, Figure S1: MAX phase unit cells. Figure S2: Differences between automatic and manually fitted *a* and *c* lattice parameters against experimental *a* and *c* lattice parameters (“fit” denotes manually fitted and “auto” denotes the automatic optimisation results). Labels for each MAX phase are shown at the top of the figure above the corresponding *a* and *c* experimental lattice parameters. Figure S3: Comparison of calculated bulk moduli against experimental values. The black dashed line is where the experimental bulk modulus is equal to the calculated bulk modulus, i.e., the line *y* = *x*. Figure S4: Charge density greyscale colour map through a (110) slice of the bulk unit cell with the Bader charge of each element. Table S1: Initial MAX phase lattice parameters and internal coordinates. Table S2: Optimized k-point grids for bulk MAX phases. Table S3: Optimized cut-off energies for bulk MAX phases. Table S4: Literature and manually fitted lattice parameters. *a* × *c* represents the *a* and *c* lattice parameters respectively.
