# Supplementary material for: DFT Study of MAX Phase Surfaces for Electrocatalyst Support Materials in Hydrogen Fuel Cells
Source: Materials (Basel). 2020 Dec 25;14(1):77. doi: 10.3390/ma14010077 (PMC7796375; doi:10.3390/ma14010077)
Supplement: Supplementary file 1 [file materials-14-00077-s001.pdf]

Supplementary Materials: DFT Study of MAX Phase Surfaces for Electrocatalyst Support Materials in Hydrogen Fuel Cells

Jonathan Gertzen [1], Pieter Levecque [1], Tokoloho Rampai [2] and Tracey van Heerden [3]*

1. Methodology

Density Functional Theory (DFT) calculations were performed using Vienna *Ab-Inito* Simulation Package (VASP). A selection of functionals (PW91(GGA), LDA, PBE, rPBE) were evaluated when determining the properties of the three bulk MAX phase materials (Ti_2AlC, Ti_3AlC_2, and Ti_3SiC_2). For all functionals the same procedure of determining bulk phase properties was followed.

1.1. Bulk MAX phase DFT calculation methodology

Bulk phase geometries were initialized using the internal coordinates described in literature [1,2]. The geometry of the systems is given in Table S1 and shown visually in Figure S1.

Table S1. Initial MAX phase lattice parameters and internal coordinates

**MAX Phase**		**Ti_2AlC**	**Ti_3AlC_2**	**Ti_3SiC_2**
a (Å)		3.040	3.075	3.0665
c (Å)		13.60	18.58	17.671
Volume $\left(\text{Å}^3\right)$		108.847	152.148	143.906
Internal Coordinates	Ti(1)	(1/3, 2/3, 0.086)	(0, 0, 0)	(0, 0, 0)
	Ti(2)		(1/3, 2/3, 0.128)	(1/3, 2/3, 0.128)
	Al / Si	(1/3, 2/3, 0.75)	(0, 0, 0.25)	(0, 0, 0.25)
	C	(0, 0, 0)	(1/3, 2/3, 0.564)	(1/3, 2/3, 0.564)

In the 312 MAX phases, Ti(1) is the inner layer of M atoms, while Ti(2) is adjacent to the A layer (see Figure S1). [1,2]

Sampling of the Brillouin zone was performed using a gamma-centered Monkhorst Pack scheme [3,4]. Gamma-centered grids are recommended for hexagonal systems such as MAX phases, as the energy convergence is significantly faster [5, s. 5.5.4]. The k-point mesh was optimized for each MAX phase system, details of which are given in Section 1.1.1.

For all bulk phase calculations, tetrahedron method smearing, with Blöchl corrections to remove the quadratic error of the tetrahedron method, was used. Electronic convergence criteria of 1×10^{-4} eV was used. For calculations where geometries were not fixed, the conjugate gradient algorithm is used to relax ions into their instantaneous groundstate, with a standard POTIM value (which controls the length of the trial step) of 0.5, and a force criteria of on each relaxed atom of <0.03 eV/Å.

1.1.1. k-point mesh optimization

The general method in the previous section was used for optimization of the k-point mesh. While optimizing k-points, a cutoff energy of 500 eV was used for the expansion of the plane waves. This cutoff energy is in excess of the recommended cutoff energies given for each element. A convergence criteria of 1 meV/atom was utilized for determining the minimum k-point grid. The converged grids for each MAX phase and functional are given in Table S2.

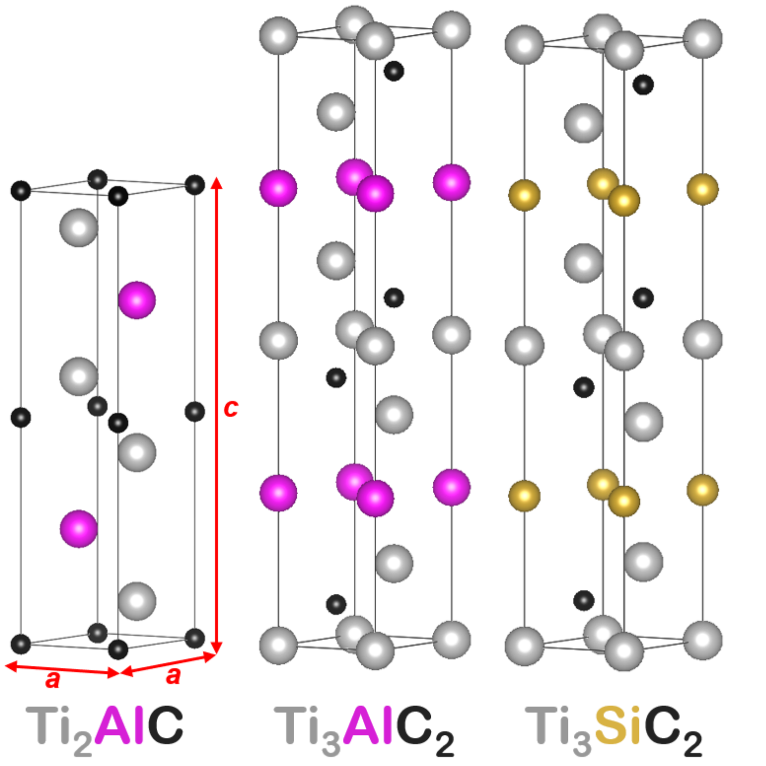

Figure S1. MAX phase unit cells

Table S2. Optimized k-point grids for bulk MAX phases

MAX phase	Functional	k-point grid	k-points in Brillouin zone	*a* k-point density (Å^{-1})	*c* k-point density (Å^{-1})
Ti_2AlC	GGA-PW91	7x7x1	8	0.0464	0.0727
	LDA	6x6x1	7	0.0552	0.0739
	PBE	6x6x1	7	0.0543	0.0727
	rPBE	7x7x2	16	0.0462	0.0362
Ti_3AlC_2	GGA-PW91	3x3x2	6	0.1077	0.0271
	LDA	7x7x1	8	0.0471	0.0546
	PBE	7x7x3	16	0.0465	0.0178
	rPBE	3x3x2	6	0.1074	0.0269
Ti_3SiC_2	GGA-PW91	3x3x2	6	0.1087	0.0279
	LDA	3x3x3	6	0.1105	0.0190
	PBE	3x3x7	12	0.1087	0.0080
	rPBE	4x4x2	8	0.0810	0.0281

1.1.2. Cutoff energy optimization

Utilizing the optimized k-point mesh described in Table S2 optimum cutoff energies for the expansion of plane waves were determined for each system. A convergence criteria of 1 meV/atom was again implemented. The minimum cutoff energies determined are given for each system in Table S3.

Table S3. Optimized cutoff energies for bulk MAX phases

MAX phase	Functional	Cutoff energy (eV)
Ti_2AlC	GGA-PW91	400
	LDA	360
	PBE	340
	rPBE	340
Ti_3AlC_2	GGA-PW91	410
	LDA	380
	PBE	340
	rPBE	360
Ti_3SiC_2	GGA-PW91	410
	LDA	370
	PBE	350
	rPBE	360

1.1.3. Unit cell / lattice parameter optimization

Utilizing the optimized k-point mesh and cutoff energies determined above, the size and shape of the unit cell was optimized. Both manual and automatic optimizations were conducted. In the manual method, the *a* and *c* lattice parameters were varied such that volumes between 90 and 110% of the volumes given in literature [1,2] are calculated. Single point energy calculations were then conducted to determine the energies of these geometries. For the automatic optimization, the ions, cell shape, and cell volume are all relaxed. The results for all three MAX phases showed consistent underestimation of the lattice parameters by the LDA functional, with all other functionals investigated generally resulted in an overestimation of the lattice parameters. The lattice parameters corresponding to the minimum energy systems are given together with experimental values in Table S4 and are compared in Figure S2

Table S4. Literature and manually fitted lattice parameters. *a* x *c* represents the *a* and *c* lattice parameters respectively

MAX phase	Literature (Å) [1,2]	Functional	Calculated (Å)
Ti_2AlC	3.040 x 13.600	GGA-PW91	3.0800 x 13.7538
		LDA	3.0200 x 13.5343
		PBE	3.0700 x 13.7623
		rPBE	3.0900 x 13.8220
Ti_3AlC_2	3.075 x 18.580	GGA-PW91	3.0950 x 18.4596
		LDA	3.0350 x 18.3278
		PBE	3.0750 x 18.6782
		rPBE	3.1050 x 18.5802
Ti_3SiC_2	3.0665 x 17.671	GGA-PW91	3.0665 x 17.9334
		LDA	3.0165 x 17.5755
		PBE	3.0665 x 17.8651
		rPBE	3.0865 x 17.8076

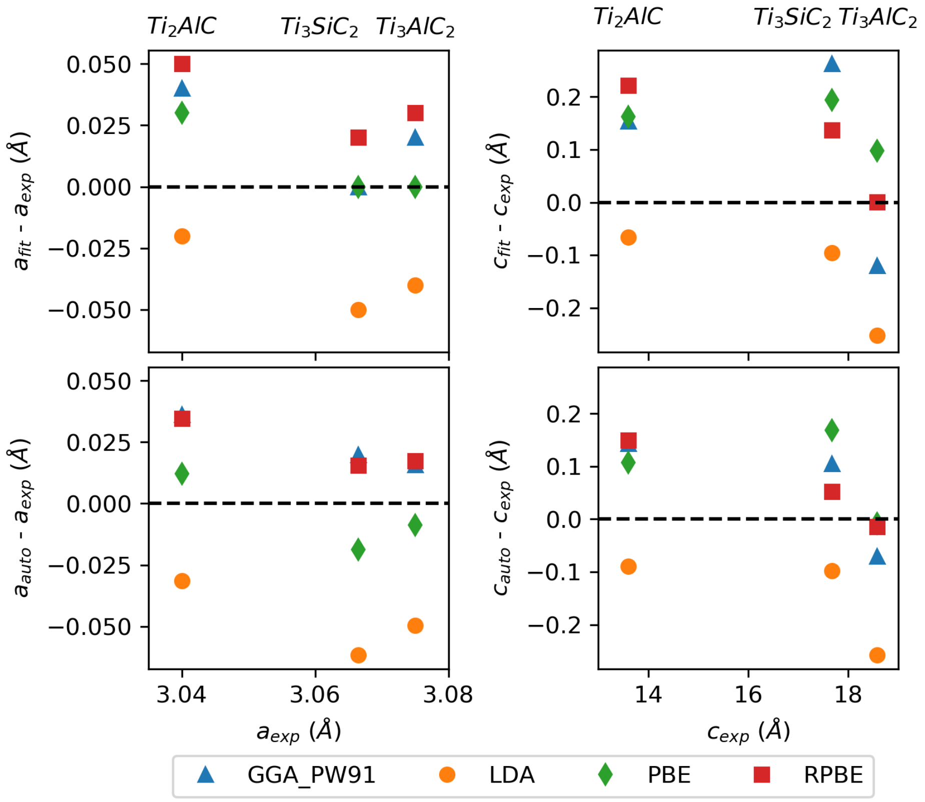

Figure S2. Differences between automatic and manually fitted a and c lattice parameters against experimental a and c lattice parameters ("fit" denotes manually fitted and "auto" denotes the automatic optimisation results). Labels for each MAX phase are shown at the top of the figure above the corresponding a and c experimental lattice parameters.

1.1.4. Mechanical properties

The predicted bulk modulus of each MAX phase for each functional can be determined by fitting the Birch-Murnaghan equation of state (EOS) [6] to the series of results generated in the manual optimization of the lattice parameters. The EOS is described mathematically by Equation 1.

$$E = E_0 + \frac{9V_0B_0}{16}\left\{\left[\left(\frac{V_0}{V}\right)^{\frac{2}{3}} - 1\right]^3 B_0^{'} + \left[\left(\frac{V_0}{V}\right)^{\frac{2}{3}} - 1\right]^2 \left[6 - 4\left(\frac{V_0}{V}\right)^{\frac{2}{3}}\right]\right\} \tag{1}$$

Where:

E = internal energy in eV
E_0 = equilibrium (minimum) internal energy in eV
V = unit cell volume in $\mathring{A}^3$
V_0 = equilibrium (at E_0) unit cell volume in $\mathring{A}^3$
B_0 = equilibrium (at E_0) bulk modulus in eV/$\mathring{A}^3$
B_0' = partial derivative of the bulk modulus with respect to the system pressure in eV/$\mathring{A}^3$

The predicted bulk modulus was calculated for each MAX phase and functional. The results are plotted against experimental values in Figure S3.

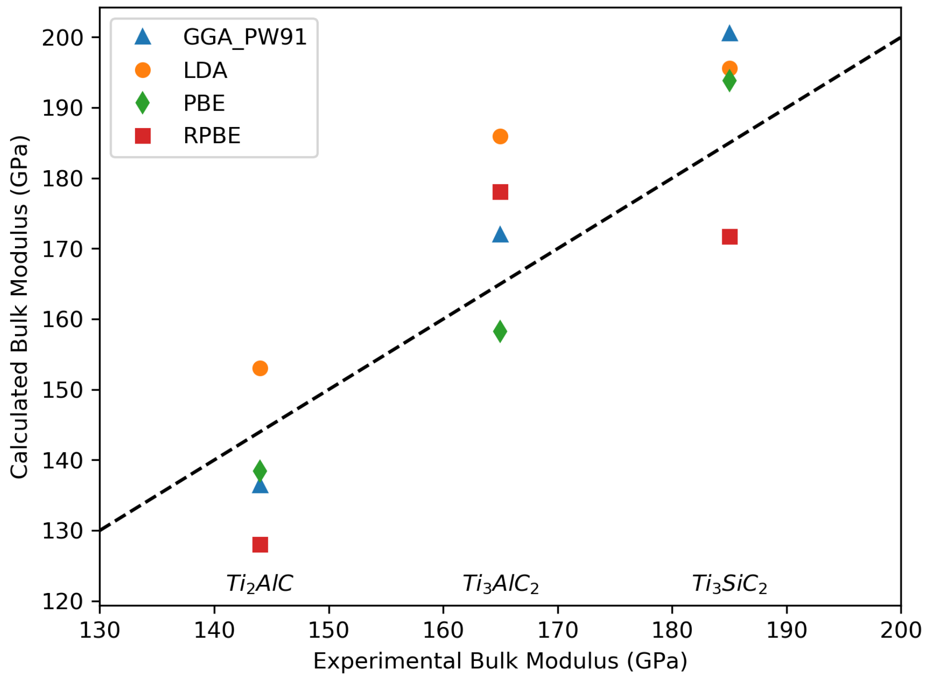

Figure S3. Comparison of calculated bulk moduli against experimental values. The black dashed line is where the experimental bulk modulus is equal to the calculated bulk modulus, i.e. the line $y = x$.

1.2. Charge Density and Bader Charge Analysis

Shown in Figure S4 is a slice through the (110) plane of the bulk unit cell. The colour map goes from white (0 $e^-/Å^3$) to black (0.25 $e^-/Å^3$).

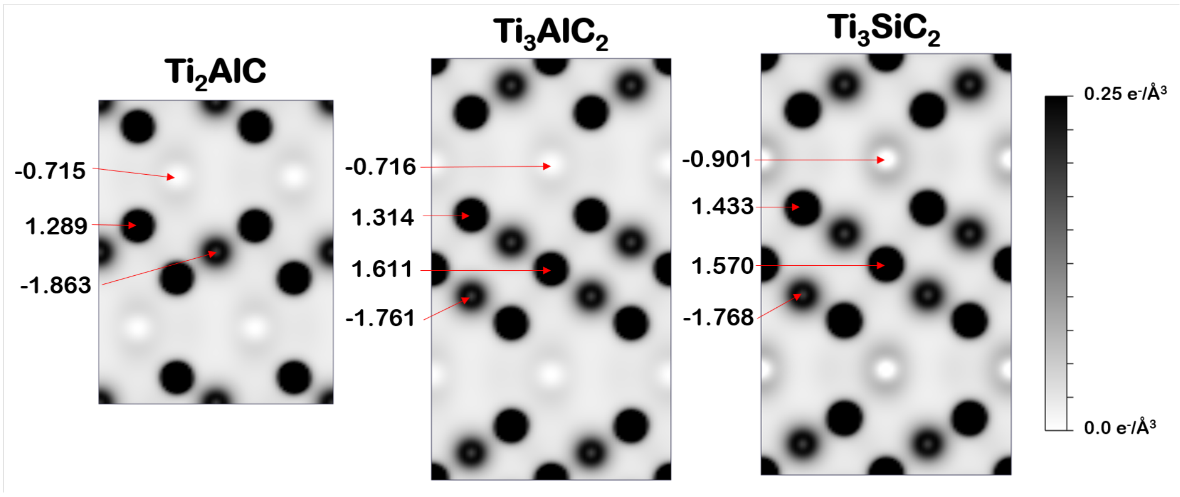

Figure S4. Charge density greyscale colour map through a (110) slice of the bulk unit cell with the Bader charge of each element

References

1. Barsoum, M.W. The $M_{N+1}AX_N$ phases: A new class of solids. *Prog. Solid State Chem.* **2000**, *28*, 201–281. doi:10.1016/S0079-6786(00)00006-6.
2. Wang, X.H.; Zhou, Y.C. Layered Machinable and Electrically Conductive Ti_2AlC and Ti_3AlC_2 Ceramics: a Review. *J. Mater. Sci. Technol.* **2010**, *26*, 385–416. doi:10.1016/S1005-0302(10)60064-3.

3. Chadi, D.J.; Cohen, M.L. Special Points in the Brillouin Zone. *Physical Review B* **1973**, *8*, 5747–5753. doi:10.1103/PhysRevB.8.5747.
4. Pack, J.D.; Monkhorst, H.J. "Special points for Brillouin-zone integrations"—a reply. *Phys. Rev. B* **1977**, *16*, 1748–1749. doi:10.1103/PhysRevB.16.1748.
5. Kresse, G.; Marsman, M. *VASP the GUIDE*; University of Vienna, 2012; pp. 1–188. https://cms.mpi.univie.ac.at/wiki/index.php/The_VASP_Manual.
6. Birch, F. Finite Elastic Strain of Cubic Crystals. *Phys. Rev.* **1947**, *71*, 809–824.

Publisher's Note: MDPI stays neutral with regard to jurisdictional claims in published maps and institutional affiliations.
